# Loss of Connectivity in Cancer Co-Expression Networks

**DOI:** 10.1371/journal.pone.0087075

**Published:** 2014-01-28

**Authors:** Roberto Anglani, Teresa M. Creanza, Vania C. Liuzzi, Ada Piepoli, Anna Panza, Angelo Andriulli, Nicola Ancona

**Affiliations:** 1 Institute of Intelligent Systems for Automation, National Research Council, CNR-ISSIA, Bari, Italy; 2 Center for Complex Systems in Molecular Biology and Medicine, University of Torino, Torino, Italy; 3 Department of Medical Sciences, Division and Laboratory of Gastroenterology, IRCCS Casa Sollievo della Sofferenza, San Giovanni Rotondo, Italy; National Research Council of Italy (CNR), Italy

## Abstract

Differential gene expression profiling studies have lead to the identification of several disease biomarkers. However, the oncogenic alterations in coding regions can modify the gene functions without affecting their own expression profiles. Moreover, post-translational modifications can modify the activity of the coded protein without altering the expression levels of the coding gene, but eliciting variations to the expression levels of the regulated genes. These considerations motivate the study of the rewiring of networks co-expressed genes as a consequence of the aforementioned alterations in order to complement the informative content of differential expression. We analyzed 339 mRNAomes of five distinct cancer types to find single genes that presented co-expression patterns strongly differentiated between normal and tumor phenotypes. Our analysis of differentially connected genes indicates the loss of connectivity as a common topological trait of cancer networks, and unveils novel candidate cancer genes. Moreover, our integrated approach that combines the differential expression together with the differential connectivity improves the classic enrichment pathway analysis providing novel insights on putative cancer gene biosystems not still fully investigated.

## Introduction

Over the past few years, cancer research has experienced remarkable advances provided by new systems biology approaches following the development of high-throughput technologies coupled to novel statistical techniques [1], [2]. One of the most used methods for the study of genetic patterns in cancer tissues is the gene expression profiling via RNAseq or microarray analysis that provides measurements of mRNA levels of the whole genetic landscape in a given biological sample. Generally, diseased tissues are compared with normal controls in order to identify groups of genes whose expression levels are significantly different in the two phenotype conditions and consequently associated to the disease [3], [4]. This population of genes, defined as differentially expressed (DE), is usually considered made of candidate biomarkers for the onset and progression of the pathology and has been widely studied for the identification of disease-related pathways [5], [6].

Although differential expression studies have been widely and successfully applied in many approaches, they present severe shortcomings in the investigation of complex pathologies. A crucial example is represented by carcinogenesis which is a multi-step process involving the gradual accumulation of genetic mutations, that can occur indifferently in regulatory or coding sites of genes. As a matter of fact, the coding region alterations and the post-translational modifications (e.g. phosporylation, acylation, methylation, etc.) can modify the protein activity without affecting the gene expression level, but altering the interaction pattern with other genes.[7]. For instance, missense and nonsense mutations in the sites coding for protein binding regions could disrupt several fundamental protein-protein interactions without modifying expression levels. A well-known case of this kind of changes in cancers involves Adenomatous Polyposis Coli (APC), which is the most common mutated gene in colorectal cancer [8], [9]. The most frequent APC mutation leads to a truncated protein that lacks the binding sites for some interacting proteins [10]. Therefore, an analysis based uniquely on differential expression studies could be ineffective for the highlighting of some key genetic drivers in neoplastic lesions. On the other hand, another crucial drawback of the differential expression analysis consists in the fact that genes are treated individually, so that interactions are not taken into account. Indeed, it is widely accepted that the comprehension of mechanisms underlying the evolution of genetic disorders like cancer must consider the contribution of interactions among genes [11]. Furthermore, it is essential to investigate the way these interactions change in the disease phenotype, with respect to the wild-type condition [12]–[14] since it is well established that not all genes are active in both states. For instance, [15] showed that in response to diverse perturbations the interaction patterns of transcription factors can be altered causing a rewiring of the network.

In the framework of gene expression profiling, the study of statistically significant correlations between gene pairs can reveal putative interactions, dependencies or coordinated activities of genes in a given biological state. In particular, networks based on gene expression pairwise correlations can represent direct gene regulations and also include genes that are indirectly connected through regulatory pathways [16]. Furthermore, since transcription is the result of a complex multi-level process, an inferred correlation network takes into account not only transcription factor-DNA interactions but also the factors that biochemically regulate the systems. Hence, it is possible to guess that modifications of interactions between genes under different experimental conditions will reflect on diverse correlation pattern outputs. In this picture, recent approaches focused on the identification of the changes in gene co-expression structures (quantified by pairwise correlations) between healthy and diseased tissues to the aim of providing better insights of altered regulation mechanisms and of indicating critical disorder driver genes [7]. In particular, differential co-expression network analyses have been widely applied and have shown important evidences for the investigation of cancer gene networks [17]–[19] and the identification of mutated but not differentially expressed genes [20].

The idea underlying the present study is that modifications of gene connectivity in biological networks are associated to significant phenotypic changes. An encouraging evidence is reported in [15] where the authors found that the connectivity of gene regulation in *Saccharomyces cerevisiae* undergoes dramatic alterations during cellular processes. Indeed, they showed that many transcription factors present only a small number of interactions retained across the different conditions, while the remaining connections are active only in specific conditions of the system.

In the present study, we show that the network connectivity can sensibly change in neoplastic tissues. As a connectivity measure of a gene we used the degree because it is a fundamental observable in graph theory and has a clear biological interpretation [15]. In particular, we studied the gene interaction changes that emerge in cancer tissues with respect to healthy controls by comparing the specific inferred co-expression networks. To this aim, we investigated on the single nodes that presented a connection structure strongly modified between two biological phenotypes. Non-parametric random permutation tests were adopted in order to highlight those gene having degree variations associated to pathology and not due to chance.

We found that a loss of connectivity in cancer networks with respect to normal ones is a common trait among the different kinds of cancer. Next, we found that a study of differential connectivity can indicate tumor-related genes not revealed by differential expression analyses. Finally, we showed how the integration of differential expression with differential connectivity can improve the classic enrichment analysis revealing pathways associated to cancer hallmarks and providing insights on novel putative biomarker systems.

## Results

We analyzed expression levels of human 339 mRNAomes including normal and neoplastic tissue samples related to five gene expression datasets of distinct neoplasias from GEO and ArrayExpress: colorectal, lung, gastric, pancreatic and cervical. All samples were profiled with Affymetrix technologies and preprocessed with Affymetrix Expression Console software (see Material and Methods).

### Loss of connectivity in cancer networks

**Figure 1:**
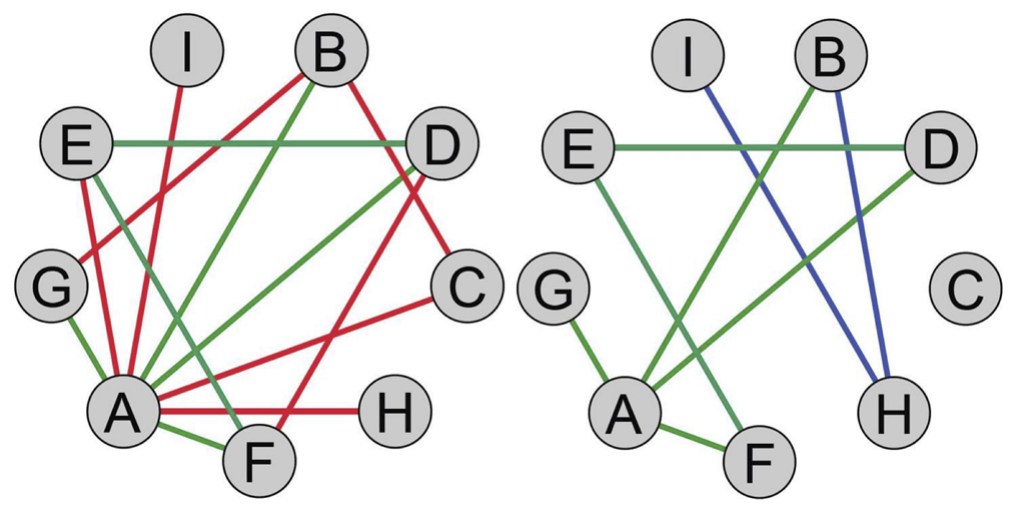
Schematic picture of connections in co-expression graphs. Healthy condition on the left and disease-affected tissue on the right. Green links remain unchanged in the two phenotypes. Red connections are loss from healthy to cancer network. Blu edges are novel connections in the cancer tissue.

We investigated the topological properties of co-expression networks in the healthy and diseased conditions in terms of gene connectivity or degree (see Material and Methods). We inferred the normal and cancer co-expression networks based on Spearman correlation coefficients: two genes were connected by an edge if the correlation coefficient between their expression profiles was not-null at the 5% significance level with a Benjamini–Hochberg [21] false discovery rate (FDR) below 20% (see Figure 1 and Material and Methods).

**Figure 2:**
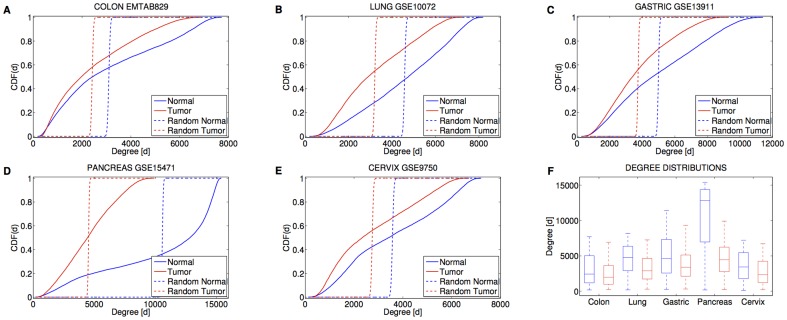
Cancer tissues are characterized by loss of connectivity. (**A-E**) Cumulative distribution functions of the gene degree. (**F**) Boxplots of the gene degrees for the five tissues in the two conditions. Red color refers to the cancer phenotype. Blue color refers to the normal phenotype. The median degree in cancer is lower than in normal conditions.

As expected, the topology of the inferred gene networks turned out to be deviated from a random model (see Figures 2A-E and Material and Methods) since their degree distributions resulted different from the ones of the corresponding random graphs with the same average degree and the same number of nodes (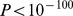, Kolmogorov-Smirnov test). Furthermore, cancer and normal networks were characterized by nodes with highly variable degrees, from genes with a few connections to ‘hubs’ with thousands of links (Figure 2A-F).

On the other side, the comparison between normal and cancer networks brought out a remarkable trait common to the diverse tumor types: co-expression gene networks of neoplastic tissues present a reduced connectivity with respect to the healthy condition (see Figure 2F). Indeed, a Kolmogorov-Smirnov test showed that all cancer networks are characterized by a gene degree which is stochastically decreased with respect to the corresponding normal graphs (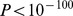).

### Gene differential connectivity and its interplay with the differential expression

The significant changes of network connectivity in cancer indicate that genes with strongly altered connections can have a role in the cancer biology and motivate a study on a connectivity-based scoring measure for the identification of putative cancer drivers. To this aim, for each gene, we evaluated the differential connectivity (DC) as difference of gene degrees in the two phenotypic conditions and we assessed its statistical significance with a p-value and a false discovery rate. Moreover, we studied the performance of this measure and its relative merit with respect to the differential expression (DE) in terms of p-value and FDR (see Material and Methods).

**Figure 3:**
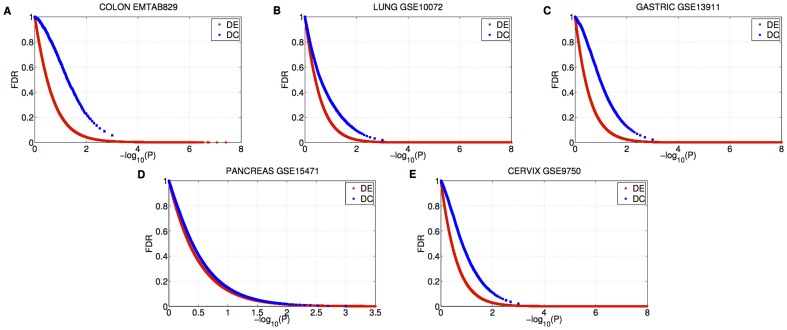
Benjamini–Hochberg False Discovery Rate (FDR) as a function of the p-value. Red color represents the differentially expressed genes (DE). Blue color represents the differentially connected genes (DC).

An analysis of FDR as a function of p-values for both differential measures showed that for p-values less than 0.01, there was a proportion of false discoveries below 22% for DC and 10% for DE (Figures 3A-E). This means that, for both measures, the number of the resulting differential features is significantly higher than would be expected by chance, although the FDR of DC is greater than the FDR of DE for each p-values.

**Figure 4:**
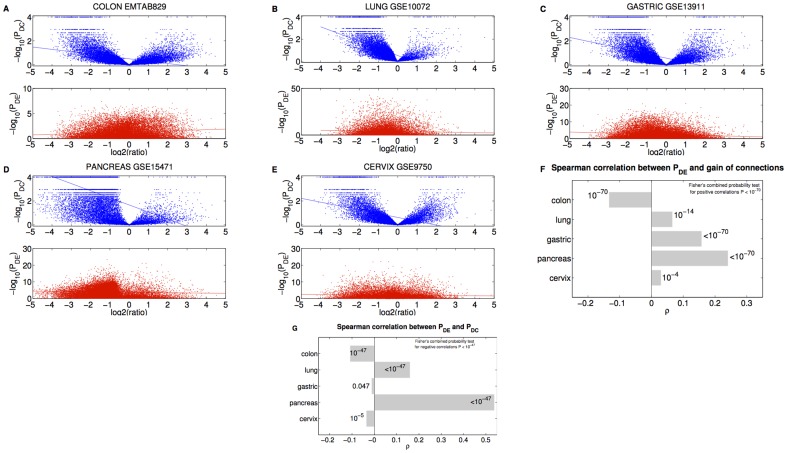
Comparison between differential expression and differential connectivity. (**A-E**) (Upper panel) Gene differential connectivity p-value 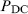 as a function of degree ratio 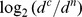. (Lower panel) Gene differential expression p-value 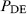 as a function of degree ratio 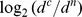. Each point represents a gene and the trend line is the least-square line. (**F**) Correlations between the differential expression p-value and the gain of connections. P-values on the bars refer to right-tail tests for the positive correlations, and left-tail tests for the negative correlations. (**G**) Correlations between differential expression p-values and differential connection p-values.

As a further investigation, we analyzed the interplay between the connectivity loss and the differential expression and differential connection p-values. We found that the smaller is the p-value of DC the greater is the number of lost connections for all datasets (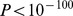, Spearman correlation, see Figures 4A-E). This result confirms the hypothesis that a significant differential connection corresponds more likely to a loss of links from normal to cancer tissues. Analogously, we found a positive correlation between the gain of degree and the differential expression p-value in every disease with the exception of colon cancer for which there is a negative correlation (Figure 4F). This indicates that differentially expressed genes are more likely characterized by a reduced degree in cancer, except in the colon case for which the differentially expressed genes tend to acquire connections in the tumor tissue (Figures 4A-F).

### Differential connectivity highlights known cancer genes

In order to investigate whether the differential connectivity can finger toward tumor-specific genes and outperforms the differential expression analysis, we collected known cancer gene lists from literature and curated databases to be tested for enrichment. Hence, the choice of a given significance threshold for DC and DE p-values turned out to be necessary. Consequently, for each cancer data set, we built two lists collecting genes having significant differential expression and connectivity at the same level of 0.05, respectively (see Table S1).

The study on the colorectal cancer data set resulted in 1870 differentially connected genes and 6792 differentially expressed genes on a number of 17400 assayed genes. The populations turned out to be distinct with 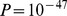 (Figure 4G). The DC gene list is enriched in tumor-suppressor genes and oncogenes commonly associated with colorectal cancer as reported in the work of [22] (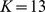, 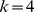, 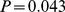) where 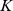 is the size of the list of known cancer genes, 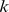 is the number of DC genes in the list and 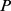 is the Fisher's exact test p-value. Adopting a more stringent level of 0.005, the DC genes are also enriched in known cancer genes from Cancer Gene Census [23] (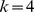, 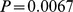, Fisher's exact test), in KEGG Disease H00020 Colorectal cancer gene list [24] (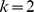, 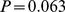) and in the genes mutated in colorectal cancers as reported in the work of Wood *et al.* [25] (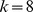, 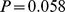). On the contrary, the differentially expressed genes resulted not enriched in the aforementioned known colorectal cancer gene lists: in [22] (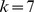, 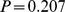), Cancer Gene Census [23] (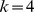, 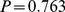), in KEGG Disease H00020 Colorectal cancer gene list [24] (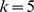, 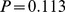) and in [25] (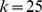, 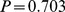).

For the lung cancer data set, the lists of genes that are differentially connected and expressed between normal and cancer lung tissues included 2749 and 7125 genes, respectively (on a number of 12157 assayed genes). Although we found a positive correlation (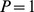) between the p-values of differential expression and the p-values of differential connectivity (Figure 4G), some remarkable exceptions, i.e differentially connected but not expressed genes, resulted critically associated to the pathology. For instance, the *EGFR* gene, resulted to be DC (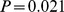) but not DE (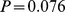), is an important frequently mutated oncogene and a drug target for lung adenocarcinoma [26], [27]. Moreover, the DC gene list is enriched in genes harboring abnormalities (mutations, amplifications and/or fusions) involved in the development of non-small-cell lung cancer as reported in the work of [28] (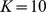, 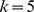, 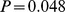). The list of these cancer genes turned out to be not over-represented in the list of the differentially expressed genes (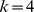, 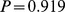).

Our analysis on the gastric cancer data set resulted in 3016 DC and 11108 DE genes (on a number of 19520 assayed genes) with the two lists significantly distinct (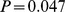, see Figure 4G). We found as differentially connected the receptor-regulated Smads (R-Smads) for TGF-beta (*SMAD2* and *SMAD3*) and for BMP signaling pathway (*SMAD1* and *SMAD5*) and the common-mediator Smad (*SMAD4*). These genes, with the only exception of *SMAD5*, are not differentially expressed (see Table S1). Moreover, we found that the lists of genes with 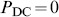 are enriched in genes that share a *TCF3*-binding site motif (E47_01, 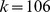, 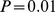) and in genes that share a *SMAD4*-binding site motif (SMAD4_Q6, 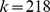, 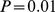) [29]. We note that it is known that TGF-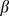-activated Smads inhibit expression of Id proteins, which in turn inhibit DNA binding of bHLH transcription factors such as E12 and E47 [30], [31]. Consequently, the differential connectivity analysis suggests alterations of the aforementioned signaling activities in gastric cancer tissues.

The study on the pancreatic cancer data set resulted in 12434 differentially connected genes and 14726 differentially expressed genes over a total of 19520 genes on the chip. Due to the large overlapping between the two groups (Figure 4G), it is not surprising that both groups are enriched for the commonly differentially expressed genes in pancreatic ductal adenocarcinoma resulting from the meta-analysis study performed on four different data sets in [32]. Moreover, the three genes (*KRAS*, *TP53*, *STK11*) associated to pancreatic cancer Omim Entry 260350 [33] were found both differentially connected and differentially expressed with the same 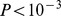. However, with a more conservative significance level of 0.005, the cancer gene list from Cancer Gene Census [23] was found to be significantly enriched in DC genes (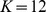, 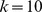, 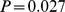) but not in DE genes (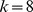, 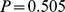).

Finally, the analysis on cervical cancer dataset revealed 2302 DC and 6186 DE genes (on a number of 12507 assayed genes) with the two populations being significantly distinct with 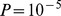 (Figure 4G). An enrichment analysis of DC and DE genes was performed on the list provided by the work of [34] made of genes commonly alterated together with those found mutated by their whole-exome sequencing study in endometrial and ovarian cancers. This study was motivated by the idea of exploiting DNA data collected from Papanicolau tests in order to reveal somatic mutations that involve the cervical tissue after being shed from endometrial or ovarian cancers. This panel of genes resulted enriched of 6 DC genes over a total of 15 (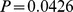, Fisher's exact test) while included 8 DE genes (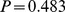, Fisher's exact test) yielding a significant overlapping only in the former case.

We outline one important remark. Although, we found that p-values for differential connection negatively correlate with ones for differential expression only for colon, gastric and cervix data sets, the overall significance of the 5 independent tests suggested that DC and DE p-values are related through a significant negative correlation (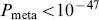, see Figure 4G). In conclusion, the DC and DE genes can behave as distinct populations and our bioinformatics analysis supports the idea that genes involved in cancer that do not change their expression can be highlighted by an analysis of differential connectivity. Consequently, one can guess that the DC genes are genes harbouring mutations that alter interactions among gene products without affecting their expression levels.

### Differential connectivity suggests novel network-based cancer biomarkers

Our study can also enlighten genes whose cancer-specific roles may be guessed from literature or are still matter of debate and further may motivate functional experiments about the involvement of these genes in the pathogenesis of the disease. From our analysis of the gastric cancer data set, the inhibitor of Bruton's tyrosine kinase (*IBTK*) resulted as the gene with the largest loss in connectivity (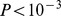) (see Table S3). As a matter of fact, the protein encoded by *IBTK* downregulates kinase activity of *BTK* which is in turn a negative regulator of Wnt-beta-catenin signaling [35]. On the other hand, the *IBTK* protein negatively regulates the activation of nuclear factor-kappa-B-driven (*NF-kB*) transcription. Since it is well established that *NF-kB* and Wnt/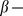
catenin signalling pathways are activated in most of gastric cancers [36], [37], it is possible to guess an involvement of *IBTK* in the evolution of tumor.

In the case of colon cancer, the second top-ranked gene for loss of connectivity with 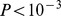 (see Table S3) is the aryl hydrocarbon receptor (*AhR*) that in the study of [38] turned out to have a crucial role in suppression of intestinal carcinogenesis by proteasomal degradation of 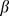-catenin, which interacts with the canonical *APC*-dependent pathway. Moreover, the sixth top-ranked gene ``deleted in polyposis 1'' (*DIP1*) has been found to have a role of tumor suppressor in colon carcinogenesis [39].

A further example comes from the lung cancer data set where the gene *TNFSF11* showed the highest loss of connectivity (see Table S3). Previous studies suggested that this protein may regulate cell apoptosis activating anti-apoptotic kinase *AKT/PKB* through a signaling complex which involves SRC kinase and tumor necrosis factor receptor-associated factor (*TRAF*) 6 (see EntrezGene Summary: [40]. Moreover, the *SRC* and *TRAF6* proteins are known to be involved in multiple aspects of tumorigenesis in human lung [41], [42]. The work of [8] confirms the involvement of *TNFSF11* in the migration of human lung tumor cells. Indeed, the gene *TNFSF11* contributes to tumor metastasis acting through *MEK/ERK*, which in turn activates *NFKB*, resulting in the activation of *ICAM1*.

As a final remark, our analysis of the lung tumor data set highlighted as the second top-ranked gene for loss of connectivity O-glycosylation initiator enzyme N-acetylgalactosaminyltransferase-14 (*GALNT14*). [50] showed that *GALNT14* may be a predictive biomarker for dulanermin-based therapy in NSCLC because they found that sensitivity to dulanermin (a protein that induces apoptosis in tumor cells) was strongly correlated with the overexpression of *GALNT14*. They also found a functional link between death receptor O-glycosylation and apoptotic signaling showing that the both pharmacologic inhibition of glycosylation and enzyme knockdown through small interfering RNAs targeting *GALNT14* reduced dulanermin-induced apoptosis [45].

These findings indicate that a differential connectivity analysis is able to detect known cancer genes and also to suggest new biomarker candidates (some potentially druggable) providing novel hypotheses for specific functional experiments.

### Differential connectivity is complementary to differential expression to reveal cancer related pathways

**Figure 5:**
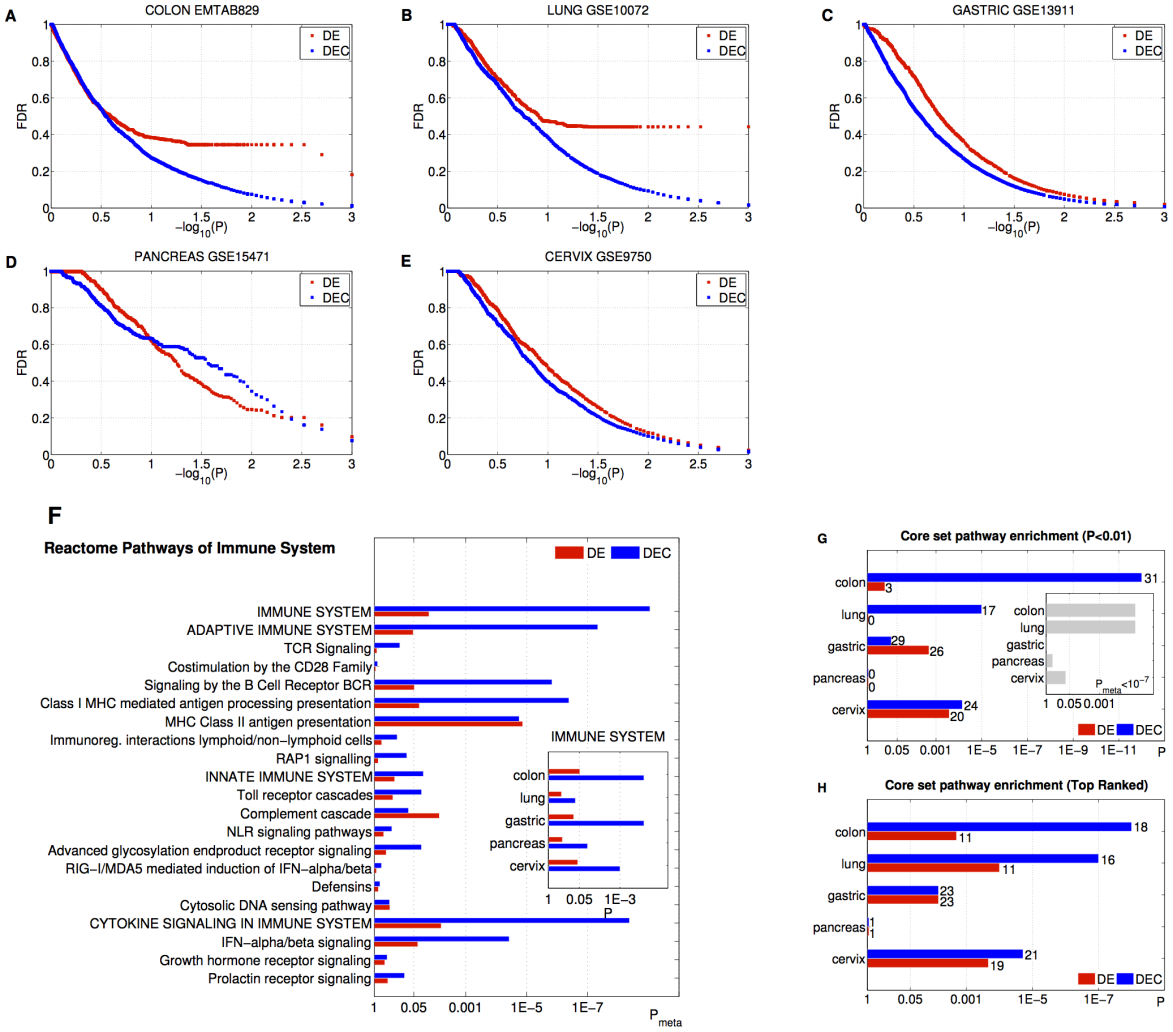
Comparison between pathway enrichment studies for differential expression and differential connectivity. (**A-E**) Benjamini–Hochberg False Discovery Rate (FDR) as a function of the p-value. Red color refers to the pathways enriched of differentially expressed genes (DE). Blu color refers to the pathways enriched of differentially expressed genes or differentially connected ones (DEC). (**F**) Reactome pathways of Immune System. Enrichment meta-analysis p-values across the tissues for ``Reactome Immune System'', its first and second sub-pathways. The histogram in the inset shows the tissue-specific enrichment p-values of ``Reactome Immune System''. (**G-H**) Core set pathway enrichment analysis. The numbers of core set pathways found as significant at 0.01 level (**G**) and in the top-ranked positions (**H**) are displayed on the bars. In the inset it is reported the p-value associated to the relative merit of DEC measure with respect to DE obtained by a permutation test.

Motivated by the evidences emerging in the previous sections, one can guess that a pathway analysis on predefined gene sets that considers both changes in gene expression and alterations in connectivity can improve the molecular characterization of disease mechanisms. For this reason, we first focused on the classic enrichment study performing a Random-Set-based pathway analysis (see Material and Methods) for the identification of pathways of functionally related genes enriched for differential expression [46]. Consequently, we investigated the pathways that resulted deregulated from a combined analysis of enrichment of genes that were either differentially expressed or differentially connected (DEC) (see Eq. 5 in Material and Methods). Our analysis tested gene lists *a priori* belonging to the canonical pathway (C2-CP) collections of Molecular Signature Database (MSigDB) [5] which collects 1452 pathways from the Reactome, KEGG, Biocarta and other databases. The behaviour of the FDR as a function of the enrichment p-value was separately studied in DE and DEC cases (see Figures 5A-E). For all cancers (except for pancreatic case), the comparison of FDRs in the two metrics showed that DE values were always greater than the corresponding DEC ones and the latter resulted less than 15% at a significance level less than 0.01. Furthermore, for colon and lung data sets, the curves of FDR resulted well separated, e.g. taking into account the differential connectivity measure yielded a 91% reduction of FDR value for a p-value of 0.003. As a consequence, we speculated that the pathways involved in tumor biology are deregulated in gene expression and characterized by altered gene interactions not necessarily affecting the expression patterns. A biological validation of this assumption required the assessment of the relative efficiency of the DEC measure in the identification of pathways underlying the general mechanisms and the tissue-specific traits of neoplasias.

In the framework of general cancer phenotype, as a specific example, we validated the two metrics in the identification of Reactome Immune System, which is related to one of the most important cancer hallmarks [46]. We performed a meta-analysis of DE and DEC enrichment combining the p-values associated to the different tissues (Fisher's combined probability test). Interestingly, the DEC meta-analysis p-value associated to Immune System (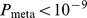) turned out to be much smaller than the corresponding DE value (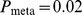) which is above the significance level of 0.01 (Figure 5F). We point out that if one considers the tissue-specific enrichment analysis, the DEC enrichment p-values always result smaller than the corresponding DE values. Moreover, the classic pathway analysis is not able to indicate, for any organ, the Reactome Immune System as significant at level of 0.05 either (see inset in Figure 5F).

On the other side, the ability of detecting organ-specific cancer traits was tested on appropriate ``core sets'' that collect known tumor-specific hallmark systems (see Material and Methods). In particular, we investigated whether DEC enrichment analysis outperforms the classic DE approach in prioritizing the pathways in the core set.

We studied separately the performances of DE and DEC measures to identify as significant the cancer core set pathways. In particular, for each measure, we assessed the significance of the number 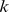 of core-set pathways having a 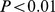 (Figure 5G) by using Fisher's exact tests. We therefore note that the exploitation of DEC genes allows to better reveal known cancer pathways. Indeed, the DEC enrichment outperforms the DE analysis both in terms of number of significant pathways and p-values for colon, lung and cervix. The difference between DEC and DE performances was assessed by a random permutation test. In particular, we compared the weighted numbers of significant core set pathways resulting from the two analyses (see Materials and Methods). Although there is a strong evidence of the relative merit of DEC analysis only in colon and lung 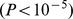 together with a slight indication for cervix 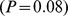, we see that considering DEC genes in pathway enrichment analysis globally unveils more signals associated to the pathology, since the overall significance is 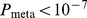 (see inset in Figure 5G).

Furthermore, we quantified how much the pathway rankings obtained with the two metrics differ from a random ordering in the identification of cancer pathways (Figure 5H). To this end, for each measure, we ranked the pathways in terms of enrichment p-values and counted the number 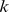 of core-set pathways present in the first top-ranked positions in the lists (see Table S2). For instance, in colorectal cancer, in the first 104 positions we found 11 core-set pathways enriched of DE genes (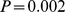) versus 18 pathways enriched of DEC genes (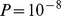). Interestingly, we note that DEC enrichment analysis always outperforms the DE measure with the exception of gastric and pancreas cases, for which the two metrics are equally efficient in the identification of the core set pathways. Indeed, the DEC approach globally ranked in higher positions cancer-related pathways than the classical DE enrichment analysis as confirmed by Fisher's combined probability test p-values show (DE 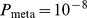; DEC 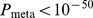).

Finally, we point out that for pancreas case, the cancer core set is unexpectedly under-represented among both DE and DEC pathways (Figure 5G-H). As a matter of fact, rather than the pathways commonly associated to pancreatic cancer, DEC analysis found more altered those pathways (see Table S2) involving the neuroactive ligand-receptor interaction and the olfactory transduction together with their superfamily of rhodopsin-like G protein-coupled receptors (GPCRs). The links between olfactory transduction and pancreatic cancer are still not clear but previous sequencing analysis of human pancreatic tumors reported many somatic mutations on the olfactory receptor genes [47]. The GPCRs are cell-surface molecules involved in signal transmission that are known to have crucial roles in tumor growth and metastasis [48]. GPCRs represent a gate through which outside signals, such as insulin, glucose, or carcinogens, may be transmitted into a cell and induce a cascade of responses related to carcinogenesis [47]. This last example demonstrates that our integrated approach that combines measures at gene-level (DE) with measures at systems-level (DEC) may enlighten novel cancer driver processes shifting the focus on mechanisms of carcinogenesis and tumor progression not still properly investigated.

## Discussion

Differential gene expression analysis is a standard technique that has been widely and successfully applied for the identification of disease biomarkers. However, it is well established that in complex pathologies like cancer, alterations in the coding regions of genes can influence their functional activities without affecting their own expression levels. In this framework, we hypothesized that co-expression network approaches based on the study of connectivity could reveal those driver genes that change their interactions without a sensible difference in expression.

For instance, consider a transcription factor (TF) 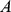 which is co-expressed with a collection 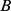 of genes in the healthy tissue. Then, suppose that in cancer tissue the TF 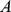 coding gene turns out to be not differentially expressed while the mutual co-expression with 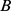 is significantly changed (e.g. some connections are removed). Although TF 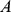 retains its normal expression level in the diseased tissue, its activity has been significantly altered and consequently its regulatory effects acting on 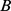 have been modified (i.e. the expression levels of genes whose promoter regions this transcription factor binds are mutated). In other words, the “rewiring” of 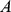 can be driven by alterations that affect the co-expression with 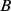, keeping unchanged the expression level of 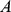. Alterations of this kind are widely known in literature [49], [50] and can occur according one of the following mechanisms: (1) Mutations in the coding region of A that lead to non-functional protein, i.e. a protein unable to bind the promoter of 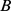; (2) Alterations in the mechanisms underlying post-translational modifications of TF 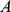 that inhibit its activity (i.e. ligand interactions, phosphorylation, acetylation, oxidation, glycosylation, etc.). Consequently, we would be not able to reveal a possible role of 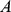 in the disease simply considering its expression levels, while from a differential connectivity point of view we could uncover its activity modifications due to the aforementioned mutations.

Motivated by these arguments, we introduced the differential connectivity on gene co-expression networks as a measure to identify candidate genes that could have a key role in cancer but that could not be highlighted by a differential gene expression analysis. In particular, we used pairwise correlations as co-expression measure to assess direct and indirect interactions between genes represented in the form of gene networks. It is important to point out that the adopted co-expression measure is able to highlight co-regulation between genes when it is mediated both by protein-coding elements present on the microarray and by non-coding elements not explicitly assayed in the network.

Our study shows that loss of connectivity in co-expression gene networks is a common trait of cancer tissues and that connectivity-based approaches can highlight novel putative cancer genes. The over-representation of known cancer genes in our findings gives a reasonable prospect that the list of top ranked genes harbours some novel tumor biomarkers not yet recognized. Importantly, in the study of gene biosystems, our connectivity-based method complements and extends the informative content provided by differential expression approaches. Indeed, we found that known pathways involved in tumor biology are enriched of genes characterized by significant alterations either in expression profiles or in their co-expression patterns (DEC).

Motivated by these findings, we suggest our integrated pathway analysis as a valid hypothesis generator for the discovery of novel cancer-related biosystems. As a matter of fact, an investigation of pathways significantly enriched of DEC genes at level of 0.05 for all cancer datasets provides a picture of the mechanisms that are commonly altered in cancer regardless the tissue type. These pathways include – among others – gene lists related to the adaptive response and the cytokine signaling in immune system (see Table S2). These are expected to be altered since evasion of cancer cells from destruction by immune cells, and tumor-promoting consequences of inflammatory responses are known hallmarks of cancer [46]. In detail, we point out that our study focuses the attention on the genes involved in MHC class II antigen presentation and on those genes that are responsible for the antiviral mechanism mediated by IFN-stimulated genes. Our findings confirm the requirement of a deeper understanding of the functions of these molecules in order to identify additional targets for new immunotherapeutic strategies that will aim to interfere with undesidered immune responses [51], [52].

In summary, the findings of the present study show a correspondence between known cancer biomarkers and differentially connected genes. Hence they yield the encouraging evidence that the biological meaning of co-expression changes can be interpreted in terms of modifications of cancer genome landscape. Consequently, a natural outlook of this work would be a rigorous biological validation that confirms the hypothesis that loss of connectivity fingers toward genes harbouring alterations (e.g. mutations, losses and deletions, promoter DNA methylation) or affected by post-translational modifications (e.g. phosphorylation, acylation, methylation, etc.) in tumours. In the future, this validation process should be possible as matched multi-dimensional data with a high number of samples for each kind of mutations will be available thanks to the research efforts in cancer systems biology.

## Materials and Methods

### Data collection

We collected five published gene expression datasets associated to five different cancer tissues for a total of 339 mRNAomes: (E-MTAB-829) 14 tumor and 14 matched normal samples of colorectal cancer from Affymetrix GeneChip Human Exon 1.0 ST; (GSE10072) 49 tumor and 58 healthy controls of non small cell lung cancer from Affymetrix GeneChip HG U133A; (GSE13911) 31 tumor and 38 healthy controls of gastric cancer from Affymetrix GeneChip HG U133Plus2; (GSE15471) 39 tumor and 39 healthy controls of pancreatic cancer from Affymetrix GeneChip HG U133Plus2; (GSE9750) 33 tumor and 24 healthy controls of cervical cancer from Affymetrix GeneChip HG U133A. Quantification of mRNAs was perfomed using Affymetrix microarrays, and the data were preprocessed using Affymetrix Expression Console Software: probe-level data in each tissue were background-adjusted, base 2 log transformed and normalized with the Robust Multi-array Analysis procedure.

### Network inference

Given 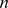 labelled samples (i.e. patients) and 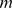 variables (i.e. genes) associated to a given phenotype, let us consider the expression profile data set represented by the 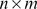 matrix 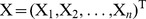 where each 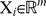. The mathematical representation of co-expressions between gene profiles can be given by a *graph* or a *network*. A graph is defined as a pair of sets 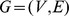 where 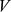 is the set of nodes or vertices (i.e. genes) and 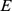 is the set of edges (i.e. non-null correlations) that join the nodes. The connection structure of the graph 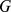 can be represented in the form of a 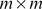 adjacency matrix **A**(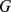), where 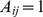 if node 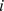 and node 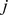 are correlated and 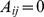 otherwise. Since the graph is undirected (i.e. links in the network do not present any direction) we set 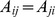, with the convention that self-loops are absent, i.e. 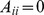.

In order to build the co-expression networks associated to the two different phenotypes, we divided each data set in a pair of subsets, called ‘normal’ and ‘cancer’, according to the corresponding label of the samples. Then, in order to take into account non-linear interactions between the variables, we considered the Spearman correlation coefficients between each pair of genes, which are equivalent to the linear Pearson correlation coefficients between ranks. For each correlation value, we evaluated the p-value for testing the hypothesis of no correlation against the alternative that there is a non-zero correlation, in the large sample approximation. The large sample approximation is based on the asymptotic normality of Spearman rank coefficient 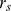, suitably standardized, i.e. for a number of samples larger than 10, in the null case the standardized version of 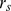, 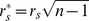, follows an asymptotic 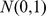 distribution [53]. In order to control the expected proportion of incorrectly rejected null hypotheses, we evaluated the Benjamini-Hochberg False Discovery Rate. Then, we set a link between two genes when the p-value was less than 0.05 and the FDR below 20%. In this way, we obtained ‘normal’ and ‘cancer’ networks for each disease where nodes are genes and links are significant not null Spearman correlation coefficients between pairs of genes.

### Gene differential connectivity in co-expression networks

The most elementary feature of a complex network is the *degree* or *connectivity* 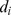 of the 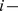
th node, that is defined as the number of edges connected to that node. Hence, it can be considered a measure of the number of vertices interacting with a certain node. The degree of a node can be evaluated in terms of adjacency matrix as
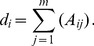
(1)


From a biological point of view, in a co-expression network the degree of the 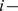
th node quantifies the amount of genes ‘co-expressed’ with the 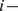
th gene. Given 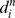 and 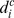 the degree of the 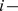
th gene in normal and cancer tissues, respectively, one defines
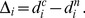
(2)


Consequently, the 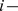
th gene is said to be “differentially connected'' (DC) when 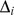 is significantly different from zero. In order to assess the statistical significance of 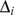, for each dataset, we randomly assigned patients to one of the two phenotypic groups and evaluated 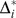 for each permutation. We repeated the shuffles 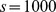 times to obtain the random distribution [54]. The p-value, 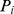, associated to 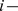
th degree difference 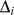 is evaluated as:
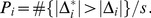
(3)


where 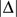 is the absolute value of 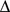 and 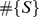 is the cardinality of set 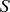. For the multiple hypothesis correction, we controlled the Benjamini–Hochberg False Discovery Rate associated to each 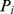 [21].

### Gene differential expression

The gene differential expression p-values were evaluated by a two-tailed Student's t-test and the p-values were controlled for multiple testing using the Benjamini–Hochberg procedure.

### Random networks

From a mathematical point of view, the analysis of degree distributions is fundamental for the classification of different topologies of networks. In order to check that inferred networks were significantly different from random graphs with the same average degree and same number of nodes, the following procedure was carried out. First, we built random graphs where each pair of nodes was connected with the elementary probability 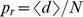 where 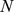 is the number of nodes and 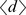 is the average degree of the real network (see dashed lines in Figure 2A-E). Then, we assessed the difference between the degree distributions relative to true and random networks by using a Kolmogorov–Smirnov test.

### Pathway enrichment analysis for differential expression and differential connectivity

In order to test the enrichment of a gene-set for differential expression, a restandardized p-value was computed using a Random Set (RS) procedure. In details, the statistical significance of the relationship of a given pathway with the phenotype is assessed with respect to two null hypotheses: the first concerns the lack of association between gene expression profiles and phenotype; the second concerns the invariance of the enrichment score with respect to the identity of the genes involved in the gene set [6]. The procedure is described in the following. Let 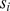 (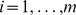) be a score associated to each gene. This score is a quantitative measure of differential expression which in our case is based on a two-sample t-statistics 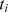, where the two samples are different phenotypes or conditions. Specifically,
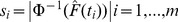
(4)where 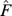 is the cumulative distribution function for a 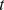 distribution having 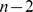 degrees of freedom, and 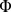 is the standard normal cumulative distribution function. Given the gene set 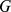 with 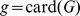, the restandardized measure of its deregulation is
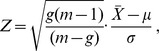
where 
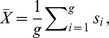
 and 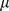 and 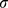 are the average and the standard deviation estimated on the full set of gene scores, respectively. Significantly large values of 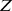 are expected if 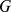 is deregulated in the experimental conditions analyzed. The p-values are computed using a phenotypic permutation test [54]. In order to test the first null hypothesis, RS method performs 1000 permutations of the sample labels and recomputes the statistic on each permuted data set. The restandardized form of the statistics is performed in order to take into account the second test.

To gain biological insights from the analysis of the differential connected or expressed genes, we searched for the signaling pathways impacted by the observed changes in terms of expression or connection patterns at gene-level. The investigation of the deregulated pathways resulting from a combined analysis involving both differentially connected and expressed genes was performed as follows. For the 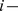
th gene, p-values of differential expression and connection were combined to form the test statistics
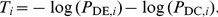
(5)


Then, for each pathway, we computed an enrichment score for the differential expression or connection (DEC) as the average of 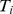 on the genes in the pathway and the score significance was assessed by permutation tests with 1000 random drawns of gene sets of the same size as the pathway [54].

For both enrichment pathway analyses, the multiple testing was controlled by applying the Benjamini-Hochberg false discovery rate (FDR) algorithm [21].

### Construction of cancer pathway core sets

We built the cancer core sets using the following procedure. The first step was the selection of the entries in the Human Disease section of KEGG Pathway collection associated to the specific cancer of interest, e.g. hsa05210 for colorectal cancer. For each entry, we collected the KEGG related pathways (e.g. MAPK signaling pathway, Cell cycle, etc.). Then, for each of them we retrieved the related pathways from the remaining databases according to specific queries on the MsigDB C2-CP collection. Finally, the resulting pathways were manually filtered and collected in a curated cancer-specific core set. The exceptions in this procedure were represented by the gastric and cervical cancer cases for which a specific KEGG pathway entry is not present. Consequently, we used the same procedure with the condition that the starting KEGG entry were ‘Pathways in Cancer’ (hsa05200) that corresponds to the most generic cancer entry.

### DEC measure validation by a permutation test

Given the p-values of the core set pathways for DE and DEC measures, we evaluated the score as
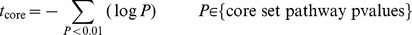
in order to obtain a weighted version of the counting of core set pathways at the significance level of 0.01. Then, we tested whether the difference 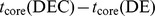 were significantly greater than the one obtained on 10000 sets of pathways, with the same size of the core set, randomly drawn from the entire collection.

## Supporting Information

Table S1 Differential gene expression and connection. Separate sheets are provided for each tissue. For each gene, the following information is reported: gene symbol, p-value DE, FDR DE, t-test statistics DE, p-value DC, FDR DC, loss of connections.
(XLSX)

Table S2 Pathway enrichment analysis for differential expression and connection. Separate sheets are provided for each tissue. For each pathway in C2-CP collection of MSigDB, the following information is reported: pathway name, p-value DE, FDR DE, p-value DEC, FDR DEC. The yellow highlighted pathways refer to the tissue-specific cancer core set.
(XLSX)

Table S3 Top ranked gene lists. List of genes having a differential connection p-value equal to zero that are ranked according the descending order of the absolute value of the degree difference.
(XLSX)
